# Histomorphometrical and CBCT Evaluation of Tissue Loss Progression Induced by Consecutive, Alternate Ligatures in Experimental Peri-Implantitis in a Dog Model: A Pilot Study

**DOI:** 10.3390/jcm11206188

**Published:** 2022-10-20

**Authors:** Lucia-Camelia Boldeanu, Marius Boariu, Darian Rusu, Adrian Vaduva, Alexandra Roman, Petra Surlin, Ioana Martu, Razvan Dragoi, Aurel Popa-Wagner, Stefan-Ioan Stratul

**Affiliations:** 1 Department of Periodontology, Faculty of Dental Medicine, Anton Sculean Research Center for Periodontal and Peri-Implant Diseases, “Victor Babes” University of Medicine and Pharmacy, 300041 Timisoara, Romania; 2 Department of Endodontics, Faculty of Dental Medicine, TADERP Research Center, “Victor Babes” University of Medicine and Pharmacy, 300041 Timisoara, Romania; 3 Department of Pathology, Faculty of Medicine, ANAPATMOL Research Center, “Victor Babes” University of Medicine and Pharmacy, 300041 Timisoara, Romania; 4 Applicative Periodontal Regeneration Research Unit, Department of Peridontology, Faculty of Dental Medicine, Iuliu Hatieganu University of Medicine and Pharmacy, 400012 Cluj Napoca, Romania; 5 Department of Periodontology, Faculty of Dental Medicine, University of Medicine and Pharmacy, 200349 Craiova, Romania; 6 Department of Dental Technology, Faculty of Dental Medicine Grigore T. Popa University of Medicine and Pharmacy, 700115 Iasi, Romania; 7 Department of Balneology, Medical Rehabilitation and Rheumatology, Center for Assessment of Movement, Functionality and Disability, “Victor Babes” University of Medicine and Pharmacy, 300041 Timisoara, Romania; 8 Experimental Research Center in Normal and Pathological Aging (ARES), University of Medicine and Pharmacy, 200349 Craiova, Romania

**Keywords:** animal study, dental implant, ligature induced, naturally occurring, peri-implantitis, histomorphometry, cone-beam CT

## Abstract

Objectives: Soft and hard tissue breakdown was histologically and radiologically assessed around implants with alternate, consecutively placed ligatures on the same edentulous dog hemimandible. The influence of ligatured implants (LI) on adjacent non-ligatured implants (NLI, as a possible naturally induced peri-implantitis) was also evaluated. Material and Methods: Three months after tooth extraction, five dental implants were placed in the dog hemimandible. Two months after abutment placement, ligatures were placed subsequently two months apart on alternate implants, while both intermediate implants were left without ligatures. Ligatures were kept in place during the entire experiment, and no plaque control measures were taken. Eleven months post-implantation, the animal was sacrificed. Undecalcified ground sections were cut, stained with Masson Goldner and MOVAT Pentachrome and evaluated by light microscopy. Soft and hard tissue loss was assessed using histomorphometric and CBCT parameters. Results: All NLI presented deep false peri-implant pockets on the oral aspect and pronounced vertical bone resorption on the buccal aspect. After 2, 4 and 6 months, during the breakdown period, more than 30% of the bone was lost in LI in all directions, while, despite immediate vicinity, NLI displayed less destruction. Intense inflammation, typical for induced peri-implantitis, was present, with similar intensity in LI as NLI, but in different parts of the lesions. Morphometry confirmed intense soft tissue inflammation, more bone resorption and higher amounts of infiltrated connective tissue in LI when compared with NLI. Conclusion: Within the limits of the present pilot study, the adequacy of the experimental dog model based on ligature-induced peri-implantitis was able to be successfully challenged by non-ligature models of spontaneously occurring peri-implant inflammation, while meeting the requirements for experimental designs with a very small numbers of animals. The influence of implants with severe peri-implantitis on adjacent implants resulted in less than expected tissue loss in the latter accession numbers.

## 1. Introduction

Peri-implantitis is an infectious disease and is considered as a common biological complication in implant therapy [1,2,3,4,5]. The clinical features of this condition include bleeding on probing, while radiographic and histopathological analysis reveal the loss of supporting marginal bone and the severe inflammation of the tissues that surround the implant [1,6,7]. According to the new Classification for Periodontal and Peri-implant Diseases and Conditions (2018), peri-implantitis was defined as a plaque-associated pathologic condition occurring in the tissue around dental implants, characterized by inflammation in the peri-implant mucosa and the subsequent progressive loss of the supporting bone [8]. While imagistic data during the loss of bone support in humans can be easily obtained [9], histopathological information about peri-implantitis can be obtained only from soft tissue biopsies and these are restricted by technical and ethical problems related to the sampling of tissues from humans [10]. In addition, the analysis of human peri-implantitis are based on small samples [11]. The animal model, in dogs especially, was considered so far as a source of accurate and comprehensive information regarding the tissue reactions in infectious peri-implant diseases [10,12,13,14].

However, the EU regulations regarding the protection of the animals used for scientific research reflect an increasing determination to reduce the number of experimental animals and their pain. If death is an endpoint of the experiments, as few animals as possible should be used [15]. Such regulations are reflected in the ARRIVE guidelines for reporting animal research, as well as outlining the desirability to use single animals as their own controls, wherever possible (“within-subject experiments”) [16,17]. 

A new approach to the ligature model in dogs, which allowed the observation that the spontaneous progression of peri-implantitis may occur after the removal of ligatures, was introduced by Zitzmann et al. [18] and was applied subsequently by other authors [1,19,20,21]. While it has been realized that ligature-induced peri-implantitis may not completely mimic the onset and progression of disease in humans, it is still generally regarded as a solely infectious model [6,22]. This results from repeated claims that bone resorption results from exposition to biofilm, not the ligature per se [23,24]. However, an increasing criticism of the ligature-induced peri-implantitis experimental model became apparent after 2000 [24,25,26,27]. To date, it remains uncertain whether the peri-implant inflammatory response is only the result of increased plaque accumulation or if the thread itself, as a foreign body, also acts as a stimulus [28].

More recently, it was suggested that the ideal canine peri-implantitis induction model would be a naturally occurring peri-implantitis induction, without the action of any ligature [25]. The literature describes only one experimental split-mouth model that tries to mimic naturally occurring peri-implantitis due to heavy, long-time, unrestricted dental plaque accumulation and so leading to an orally compromised canine model [26]. So far, there are no modified experimental designs including non-ligated implants alternated with ligated implants, nor a comparison against the consecrated model which uses exclusively ligatures.

Site-specific diseases, like peri-implantitis, are often attributable to local predisposing factors. Local contributors that can cause plaque-associated peri-implantitis include, among others, soft and hard tissue characteristics, surgical procedures, prosthetic factors and local precipitant factors (such as residual submucosal cement and dental floss) [29,30,31]. Local factors that lead to peri-implantitis also include the periodontal health of the teeth adjacent to the implant [32,33,34,35]. Furthermore, recent studies have demonstrated that the number of implants is associated with peri-implant diseases [36,37]; however, no study investigates the influence of experimentally induced peri-implantitis on adjacent non-ligated implants. 

An unanswered question remains whether implants affected by peri-implantitis influence adjacent implants. Furthermore, in an experimental design, the extent to which ligature-induced peri-implantitis mimics a clinical situation may influence their non-ligated neighboring implant and even provoke peri-implantitis still remains unknown.

Cone-beam computed tomography (CBCT) has been stated to be advantageous for the diagnosis of peri-implantitis defects [38] because it offers three-dimensional pictures and enables statements regarding the structural architecture of the bone [39]. Additionally, certain experimental ligature-induced peri-implantitis defects studies indicated that sagittal CBCT images can be correlated with the corresponding histological sections of the analyzed specimens [38,40]. Moreover, while histological sections usually result exclusively in sagittal images, CBCT can additionally provide the data of bone losses both in sagittal and mesio-distal planes, thus complementing the histology regarding the bone losses.

As the peri-implantitis problem remains today largely unresolved, emerging new therapies try to address the disease; however, in order to make their clinical application efficient, validated experimental models that closely reproduce the pathophysiology of human peri-implantitis must be designed. From this perspective, the principal aim of this pilot study was the histomorphometrical and CBCT assessment of tissue loss progression in peri-implantitis induced by ligatures placed consecutively on alternate implants on the same edentulous dog hemimandible. The influence of ligatured implants on adjacent non-ligatured implants was also evaluated.

## 2. Materials and Methods

To minimize the number of experimental animals to be sacrificed, this pilot study was conducted in a single animal, so that a maximum of data could be obtained from the experimental combination of implant positions and ligature placement.

### 2.1. Implants

The endosseous dental implants (OT medical GmbH, Bremen, Germany) consisted of 5 titanium cylinders (3.3 mm, two 3.8 mm and two 4.1 mm in diameter, all 8 mm in length), etched homogenous implant surface from titanium grade 4 with a titanium plasma spray coating applied under vacuum conditions and a 1 mm highly polished collar. Highly polished titanium abutments were connected 3 months after insertion. 

### 2.2. Animal Housing

The dog was housed under good general conditions in a single kennel with indoor and outdoor areas. The room temperature range was approximately 18 °C, with a humidity above 30%. The dog was fed once a day using a granulated dog food and water ad libitum. The maxilla was intact, without any general occlusal trauma, oral viral or fungal lesions. Clinical examination determined that the dog was in good general health, with no systemic involvement [41,42].

### 2.3. Surgical Procedures

The experiment was performed on one ten-years old adult half-breed dog, with a fully erupted permanent dentition (male, body weight 20 kg). All operative procedures were carried out under general anesthesia (intravenous Diazepam 0.5%, 0.4 mg/kg I.V. and Ketamine 10%, 10 mg/kg I.V., endo-tracheal intubation 2–5% isoflurane gas). To maintain hydration, the animal received a constant-rate infusion of Ringer’s solution while anesthetized. The protocol of the whole experiment was approved by the Ethical Commission of Scientific Research of UMFVBT (approval Nr. 06-16/2019). The experiment was finalized in December 2019. 

**Figure 1:**
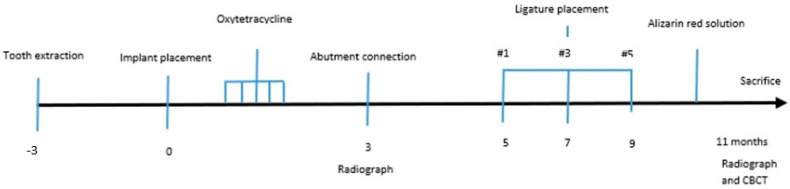
Timeline of the experiment.

**Figure 2:**
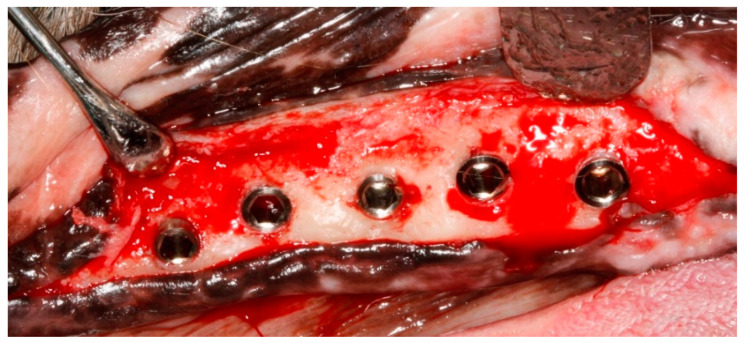
Intra-surgical image after the insertion of the implants. Note the integrity of the buccal bone, as a consequence of the rigorous prosthetic-driven implant placement.

According to the timeline of the experiment (Figure 1), the premolars and the first molar were carefully extracted after reflection of full-thickness muco-periosteal flaps from the posterior mandibular region on the right side. The teeth were hemisected at the bifurcation using a tungsten carbide bur so that the roots could easily be removed using elevators and forcipes without damaging the bony walls. The wound margins were stabilized with a continuous suture and allowed to heal. Three months later, the five endosseous implants were inserted in the edentulous regions using standard instruments and surgical techniques. Following crestal incision, a full-thickness muco-periosteal flap was elevated to expose the site for implant placement. The interdental bone and any other bony prominences were trimmed with a large round bur and with Buser bone scrapers (HuFriedy, Chicago, IL, USA) to facilitate healing. The implant beds were drilled by standard drilling procedures, in accordance with the manufacturer’s instructions, under an abundant cooling stream of sterile 0,9% physiologic saline. The gradual widening of the implants site was obtained using a series of stainless-steel drills. The implants were placed in an increasing diameter mesio–distal order (implant No. 1 being the most mesial and implant No. 5 the most distal, adapted to the width of the residual edentulous crest), with the coating levels coinciding with the alveolar crest of the edentulous jaw region, with a minimum distance of at least 3 mm between them (Figure 2). Care was taken to align the implants as much as possible along the same sagittal plane, closer to the buccal aspect of the bone, in a prosthetic-driven position that simulates a real clinical situation. Parallelism was ensured by using dental implant parallel pins. The flaps were replaced above the submerged implants using 5-0 non-resorbable Polypropylene sutures (Perma Sharp, HuFriedy, Chicago, IL, USA). All procedures were carried out under the supervision of two veterinary surgeons, one of them in charge of the anesthetic procedures. Healing was evaluated weekly, and the dog was fed a soft diet for 14 days after the sutures were removed.

### 2.4. Postsurgical Procedures

To be used in further research, intravital polyfluorochrome labeling was carried out for the histological evaluation of the bone reactions through the intraperitoneal injection of oxytetracycline (OXY L.A. INJ, Dopharma BV, Raamsdonksveer, Holland), 50 mg/kg body weight, every tenth day, from implant placement until abutment installation, and alizarin red S 1% (Merck KGaA, Darmstadt, Germany), 40 mg/kg body weight, 10 days before sacrifice.

**Figure 3:**
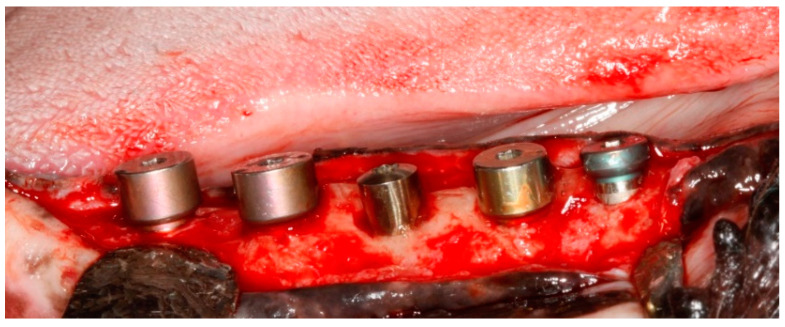
Intra-operative view of the implants at the exposure time, 3 months post-implantation, with the healing abutments in place.

**Figure 4:**
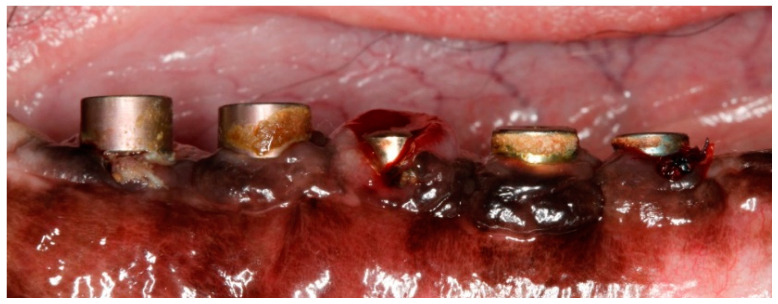
Post-operative image at 9-month post-implantation. Note the ligatures placed in alternating implants 1, 3 and 5, and the subsequent local inflammation.

Implants were exposed 3 months post-implantation and their healing abutments were connected (Figure 3). The flaps were adjusted around the titanium abutments with interrupted sutures. No oral hygiene regimen was administrated during this period, so that the peri-implant inflammation could initiate spontaneously. Five months post-implantation (two months post implant exposure), to accelerate the progression of the initial lesions, cotton ligatures were placed according to the method described by Lindhe et al. [23] in a submarginal position around the neck of implant No. 1, two months later at implant No. 3 and, after another two months, at implant No. 5 (Figure 4). Two intermediate implants (No. 2 and No. 4) were left without ligatures. The ligatures were not replaced or removed during this experiment. The animal was then fed a soft diet to induce plaque accumulation and to provoke peri-implant inflammation and the loss of bone.

### 2.5. Radiological Follow-Up

**Figure 5:**
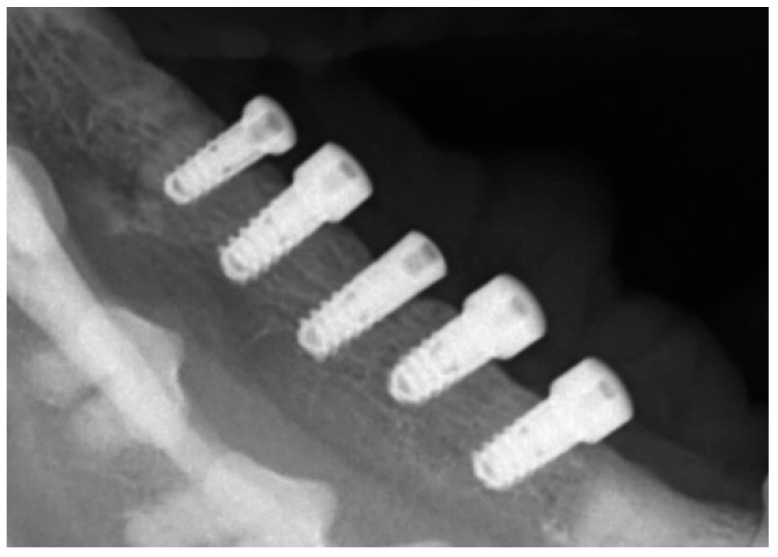
Radiological follow-up immediately after the abutment connection, revealing no post-implantation pre-exposure bone loss around the screws.

To check the peri-implant vertical alveolar bone status on the right side of the lower jaw at baseline, a conventional dental X-rays was taken immediately after the titanium abutments were installed, 3 months post-implantation (Figure 5).

### 2.6. Specimen Preparation

At the end of the experiment, 8 months after abutments were installed, the dog was sacrificed under general anesthesia (sodium pentobarbital: 200 mg/kg i.v.). The jaw was sectioned at the midline, a segment of mandible, including the implants and ligatures in situ (8 cm × 4 cm), was separated and fixated in 10% neutral buffered formalin until laboratory processing. The entire segment underwent CBCT analysis. 

### 2.7. CBCT Analysis

The jaw segment underwent CBCT evaluation (CRANEX^®^ 3Dx, SOREDEX, Tuusula, Finland). The exposure settings were 12 mA and 89 kVp, with an acquisition time of 6.1 s. The scanning parameters were set to a volume size of 80 mm height and 80 mm width, a cross interval of 0.2 mm and a panorama interval of 0.3 mm. The measurements were performed under standardized conditions using the imaging software version OnDemand3D Dental 1.0.10.7462. 

Two clinicians experienced in the evaluation of CBCT reconstructions examined the obtained images. They were blinded to the specific experimental settings and worked under standardized conditions. These conditions included computer hardware, which was technically approved for radiological observations, and working in a room equipped with window shades and dimmable light allowing a standardized low-light ambience illumination. The computer used was an HP ProBook 450 G3 with 8.0 GB RAM. The data were displayed on a monitor 39.6 cm in diagonal and at a resolution of 1920 × 1080 pixels (Hewlett-Packard Development Company, L.P., Palo Alto, CA, USA). 

**Figure 6:**
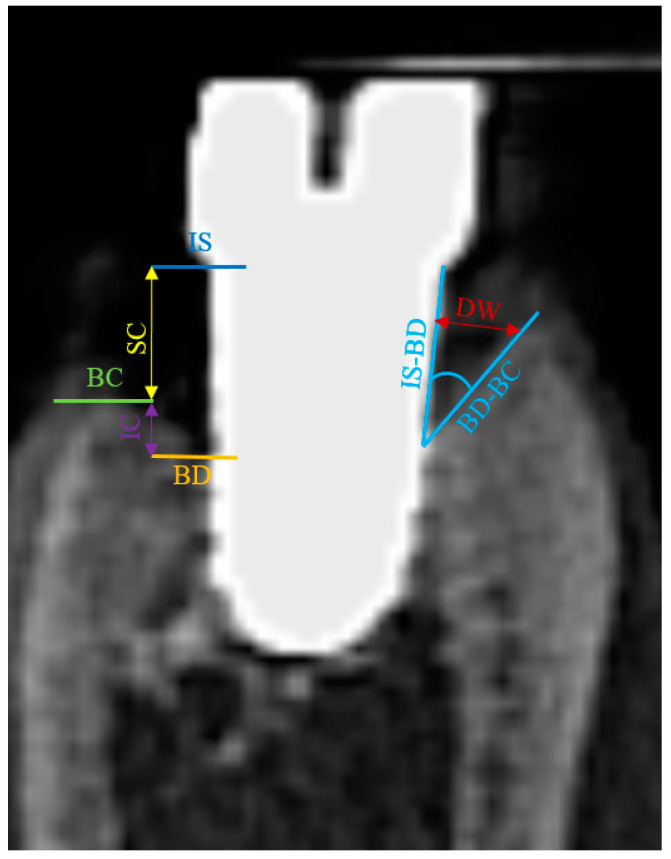
Landmarks identified in each sagittal image at all buccal, oral, mesial and distal aspects: IS (implant shoulder), the bottom of the bone defect (BD), the alveolar bone crest (BC) and the angle between segments IS-BD and BD-BC. CBCT implant #5 (original magnification ×8).

The following landmarks were identified in each sagittal image at all buccal, oral, mesial and distal aspects: IS (implant shoulder), the bottom of the bone defect (BD) and the alveolar bone crest (BC) (Figure 6). At both aspects, linear measurements were made by drawing a vertical line, following the long axis of the implant: from IS to BC (representing the supracrestal component of the defect-SC) and from BC to BD (representing the intrabony component of the defect-IC). In horizontal direction, where present, the defect width was measured as the linear distance from the implant surface to the alveolar bone crest, at buccal, oral, mesial and distal aspects.

The angulation of the peri-implant defects, as an additional measure of the extension of the bone destruction, was evaluated as well at the buccal, oral, mesial and distal aspects of the implants and represented the radiographic angle between the IS-BD and BD-BC segments. 

### 2.8. Histological Preparation of the Specimens

Technovit 9100 (Kulzer GmbH, Hanau, Germany) embedding resin was used for the infiltration and embedding steps. Sections were prepared using the cutting/grinding method described by Donath and Breuner [43] on a cutting/grinding system (Exakt, Norderstedt, Germany). A cut was performed in buccal–lingual direction through the middle of each implant on the long axis. For each implant, one section was carried out distally from the initial mid-section, while another section was carried out mesially, both on the long axis, so that two relatively equal central sections resulted. Each of these two sections were thinned and mechanically micro-polished, stepwise, using a Micro grinder machine (Exakt 400 CS, Norderstedt, Germany) reaching the final thickness of approximately 30 µm. For the staining of the sections, the deplastination of the thin-sections was performed by incubation in two baths of acetone and twice in methoxyethyl-acetate (MEA). One section of each implant sample (Section A) was stained with Masson Goldner Anilin blue (Morphisto, Frankfurt am Main, Germany) (MGA) and the other section (Section B, respectively) with MOVAT Pentachrome (after Verhöff) (Morphisto, Frankfurt am Main, Germany) (MOV). Finally, the stained slides were dehydrated and cover-slipped with mounting medium. MGA highlights the connective tissue (blue) with the differentiation of cytoplasm, muscle, erythrocytes and fibrin (red), as well as the staining of cell nuclei (black-brown), basal membranes (blue) and elastin (light red). MOV indicates collagen (light yellow) is differentiated from young osteoid (dark yellow) and osteon structures (reddish). Muscle tissue, cytoplasm and elastic fibers (red), as well as cell nuclei (blue-black) are shown differentiably.

The stained thin sections were scanned on a Zeiss Axio Scan Z.1 system (Carl Zeiss Microscopy GmbH, Jena, Germany) at 20× magnification. These images were used for morphometric measurements using the Icy Bioimage Analysis software v2.4 [44]. All sections were examined under light microscopy. Histological measurements were performed by one experienced investigator (AV). Prior to the start of the analyses, a calibration procedure was initiated for the investigator and revealed that the repeated measurements of different histological sections were similar at a >95% level.

### 2.9. Histomorphometrical Analysis

**Figure 7:**
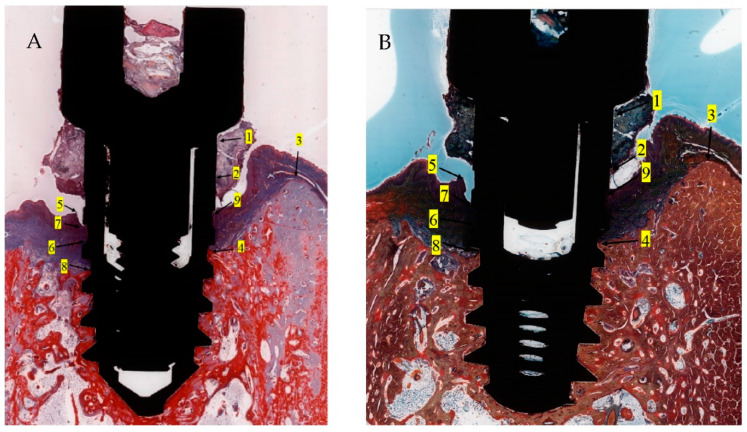
Histomorphometric landmarks identified on both buccal and lingual aspects in each implant: 1—implant shoulder (IS); 2—implant surface at the level of the bone crest (imp); 3—bone crest, defined as the most coronal point of the bone (BC); 4—bottom of the bone defect (BD); 5—margin of the peri-implant mucosa (PM); 6—the apical termination of the junctional epithelium (aJE); 7—the coronal level of the infiltrated connective tissue (cICT); 8—the apical extension of the infiltrated connective tissue (aICT); 9—the most apical extension of the submarginal biofilm that was interposed between the implant and the pocket epithelium of the peri-implant mucosa (aPlaque). (Implant #5, mirroring sections, (**A**)—Masson Goldner Anilin blue stain, (**B**)—Movat Pentachrome stain, original magnification ×2).

The following histomorphometric landmarks were identified in both the buccal and lingual aspects in each implant (Figure 7): implant shoulder (IS); implant surface at the level of the bone crest (imp); bone crest, defined as the most coronal point of the bone (BC); bottom of the bone defect (BD); margin of the peri-implant mucosa (PM); the apical termination of the junctional epithelium (aJE); the coronal level of the infiltrated connective tissue (cICT); the apical extension of the infiltrated connective tissue (aICT); the most apical extension of the submarginal biofilm that was interposed between the implant; and the pocket epithelium of the peri-implant mucosa (aPlaque).

Linear measurements of both the buccal and lingual aspects of each implant were made between: the implant surface (imp) and the alveolar bone crest (imp-BC); the implant shoulder (IS) and the level of the bone crest (BC) (IS-BC); the implant shoulder (IS) and bottom of the bone defect (BD) (IS-BD); the bone crest (BC) and the bottom of the bone defect (BD) (BC-BD); the level of the bone crest (BC) and the apical extension of the infiltrated connective tissue (aICT) (BC-aICT); the margin of the peri-implant mucosa (PM) and the level of the bone crest (BC) (PM-BC); the margin of the peri-implant mucosa (PM) and the implant shoulder (IS) (PM-IS); the margin of the peri-implant mucosa (PM) and the apical cells of the junctional epithelium (aJE) (PM-aJE); the margin of the peri-implant mucosa (PM) and bottom of the bone defect (BD) (PM-BD); the margin of the peri-implant mucosa (PM) and the apical extension of the infiltrated connective tissue (aICT) (PM-aICT); the apical termination of the junctional epithelium (aJE) and bottom of the bone defect (BD) (aJE-BD); and the most apical extension of the submarginal biofilm that was interposed between the implant and the pocket epithelium of the peri-implant mucosa (aPlaque) and bottom of the bone defect (BD) (aPlaque-BD).

The following angles, measuring the buccal and oral extension of the bone destruction, were defined: the angle between the IS-BD and BD-BC segments on the buccal aspect and the angle between the IS-BD and BD-BC segments on the oral aspect. The area of the infiltrated connective tissue (ICT) was identified and traced using a mouse cursor.

### 2.10. Statistical Analysis

Using the implant as the statistical unit, the mean values and standard deviations were calculated separately for ligated implants (LI) and non-ligated implants (NLI). Histological measurements were performed on buccal and oral aspects for each implant and CBCT measurements were performed on the buccal, oral, mesial and distal aspect of each implant. The data rows were examined using the Shapiro–Wilk test for normal distribution. Due to the small sample size, a Wilcoxon test was performed only to compare the differences between the CBCT and histomorphometric results. A *p*-value of less than 0.05 was considered statistically significant.

## 3. Results

During the experimental peri-implantitis, none of the five implants failed. Heavy plaque accumulation was observed around all healing abutments, and the peri-implant tissues became inflamed and bled, while implant No. 3 exhibited suppuration. After 2, 4 and 6 months, during the phase of experimental peri-implantitis (“breakdown period”), more than 30% of the bone was lost in LI, as shown by the CBCT images. However, NLI displayed less bone loss according to the design of the experiment. The Wilcoxon test for the differences between the histometric measurements with CBCT correspondents (SC, IC, DW, the angle made by IS-BD and BD-BC, all of which were measured buccally and orally) was not statistically significant (*p* = 0.277 > 0.05) for global measurements.

### 3.1. Histological Observations

**Figure 8:**
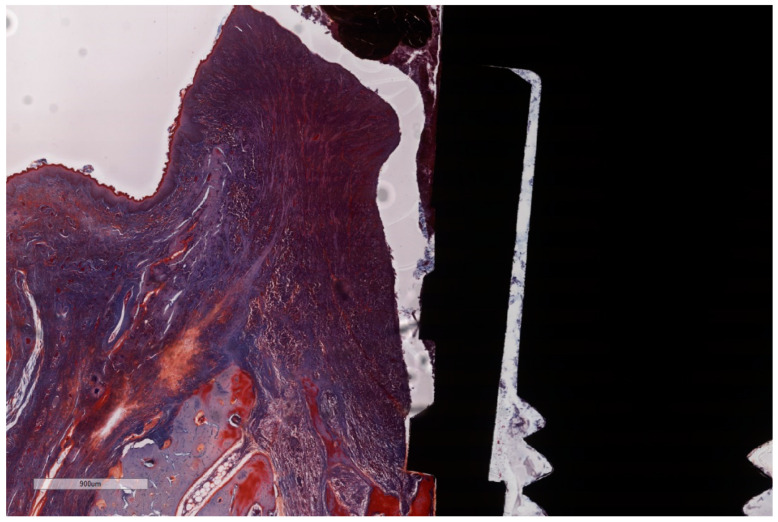
“Open wound” aspect of the ICT in contact with the implants surface and a total lack of the epithelial lining. ICT is diffusely present in the soft tissue and is seen to progress into the bone marrow, without any sign of fibrous encasement. The peri-implant sulcus is deep and the intimate contact between the soft tissues and the implant is below the BC level (implant #3, MGA, bar measure is 900 µm).

**Figure 9:**
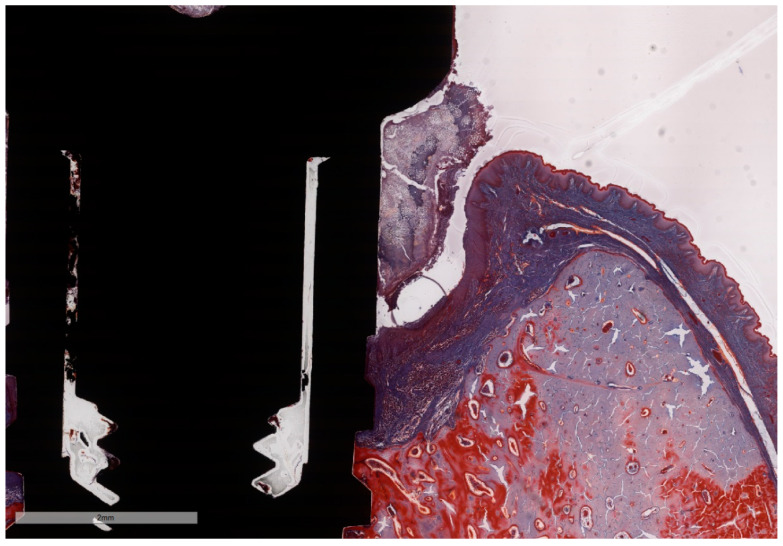
Despite the presence of intense inflammation induced by the ligature, the deep peri-implant sulcus presents a continuous epithelial lining. The intimate contact between the soft tissues and the implant is below the BC level (implant #5, MGA, bar measure is 2 mm).

**Figure 10:**
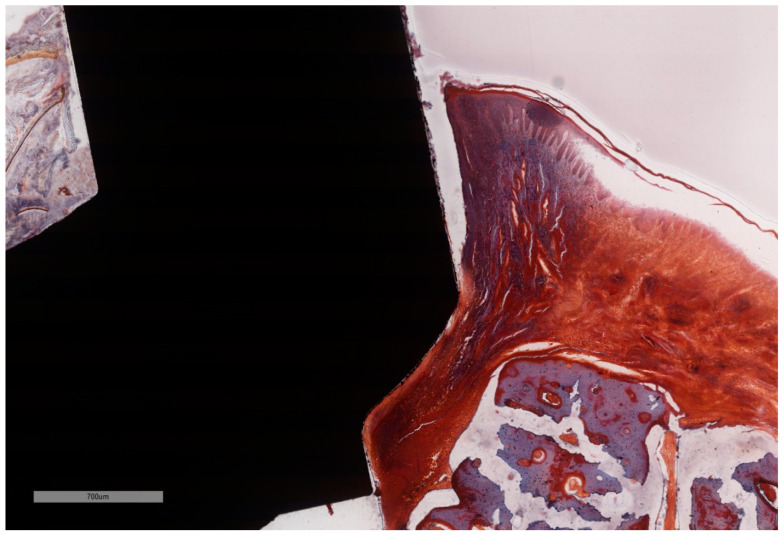
In the absence of the ligature, full epithelial lining is observed along the interface with the implant, with no ulceration. Note the intimate contact between the soft tissues and implant surface that coronally ends above the level of the BC, while the ICT remains above the BC-BD line. (implant #2, MGA, bar measure is 700 µm).

**Figure 11:**
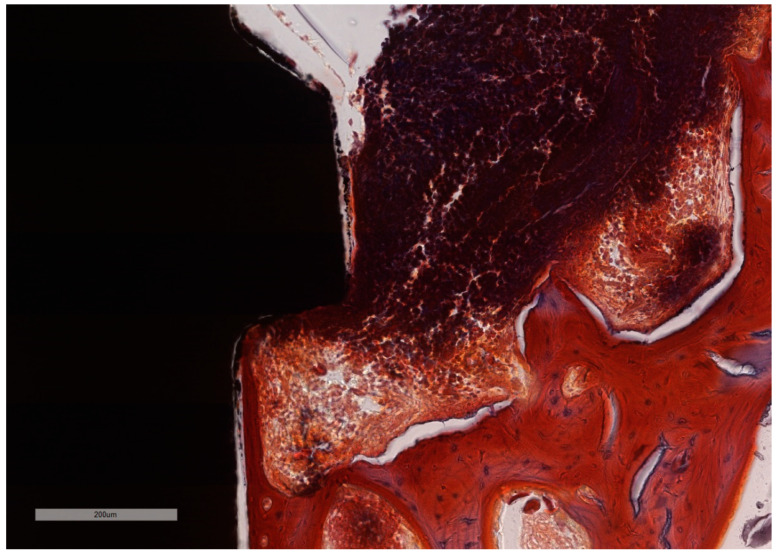
Bone in active phase of remodeling. ICT in simultaneous contact with the bone and with the implant surface with no squamous epithelium covering. Additionally, active bone remodeling is identified by the scalloped endosteal surface (Howship’s lacunae) (implant #1, MGA, bar measure is 200 µm).

**Figure 12:**
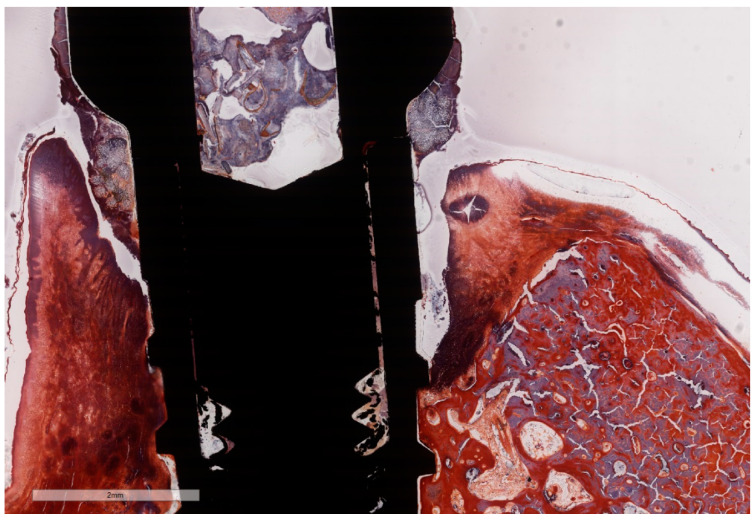
Position and effect of the ligatures. Note the intense inflammation on the left (buccal) aspect and the accentuate resorption of the buccal plate, up to its total loss. In the oral aspect, the peri-implant sulcus is deep and wide and the implant is in intimate contact with the ICT in just a limited area above the BD (implant #1, MGA, bar measure is 2 mm).

**Figure 13:**
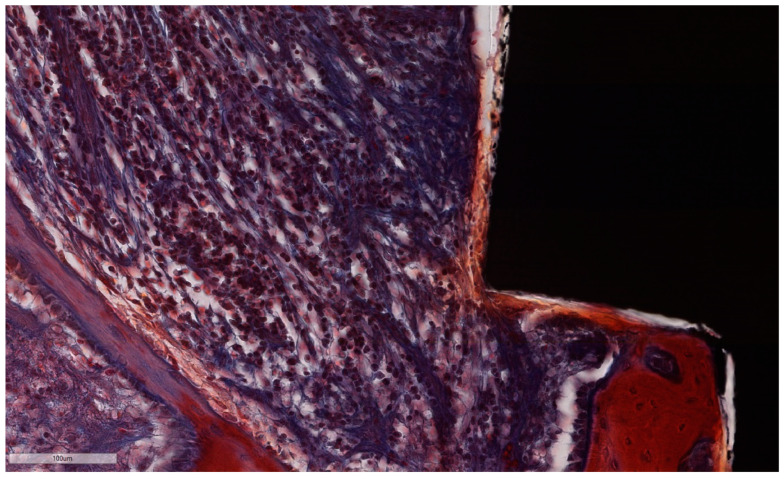
ICT in direct contact with the implant surface, without an interposed epithelial lining. Note the abundant lympho-plasmocytic infiltrate, which progresses inside the bone marrow, the thin bone trabeculae left after remodeling along with prominent osteoblast rim (implant #3, MGA, bar measure is 100 µm).

The histological assessment of the processed specimens showed the partial osseointegration of the implants in all cases. Implant and screw integrity, along with the persistence of ligatures was observed in specimens 1, 3 and 5. The bone height level was lower on the buccal aspect than on the oral aspect, the difference being bigger in the specimens with experimentally induced peri-implantitis. The peri-implant sulcus is deeper in all ligated specimens than in non-ligated ones (Figure 8 and Figure 9), and the intimate contact line between the soft tissues and the implant is seen only below the level of the BC. Epithelial lining integrity (as seen in Figure 10) was broken in several places in 2/3 of implants with ligation and in ½ of the implants without ligation, more pronounced on the oral aspect (Figure 11). In these areas of integrity loss, the inflammatory cells were identified in direct contact with the implant (Figure 12 and Figure 13). In ligated specimens, the ligatures were seen partly covered in two thirds of the cases by the soft tissues, while in one third of the implants, in the oral aspect, the ligatures were identified as below the level of the PM. The infiltration of the bone was seen in all cases of ligation and in half of the implants without ligation. In all implants, ICT was mostly composed of inflammatory cells (lymphocytes and plasma cells, with scattered neutrophils) separated by hyperemic blood vessels and a network of sparse collagen fibers. At implant No. 4, although the ICT seemed to be partially surrounded by dense collagen bundles, the deeper infiltration of the bone marrow by ICT was observed.

### 3.2. Histometric Findings

Due to the particularities of the MGA and MOV stainings, some measurements succeeded better on MGA than on MOV sections (Figure 7A,B). Moreover, artifacts resulting from the cutting–grinding process necessitated the complementing of measurements on both stainings in order to obtain values as accurate as possible.

The results from the histometric measurements are reported for LI and NLI in Table 1. Most of the bone loss was found around LI 1, 3 and 5 in all four aspects, shaped as crater-like (saucer-shaped) osseous defects. Bone loss around NLI 2 and 4 was present with a strong vertical component on the buccal aspect (mean value IS-BD = 1.26 mm), while in the lingual aspect the bone loss was negligeable, both vertically (mean value IS-BD = 0.31 mm) and horizontally (mean value imp-BC = 0.71 mm). 

The mean horizontal distance of the implant surface and the alveolar bone crest (imp-BC) was 1.70 ± 0.56 mm for LI and 0.39 ± 0.38 mm for NLI, describing, as expected, a wider bone destruction in LI. The corresponding mean vertical distance between the implant shoulder and the level of the bone crest (IS-BC) was 1.78 ± 1.31 mm for LI and 0.71 ± 0.63 mm for NLI, meaning that LI also exhibited more vertical bone loss. The mean distance between the implant shoulder and the bottom of the bone defect (IS-BD) was 3.12 ± 0.58 mm for LI and 0.79 ± 0.55 mm for NLI, illustrating, once again, an advanced bone loss around LI. Similar mean distances for IS-BD have been found in buccal and oral aspects on LI (3.49 mm, 2.89 mm, and 3.16 mm, respectively).

The mean distance between the bone crest and the bottom of the bone defect (BC-BD) was 1.33 ± 0.85 mm for LI and 0.33 ± 0.39 mm for NLI. 

Several distances between bony landmarks and soft tissue modifications have been noted. The mean distance between the bone crest and the apical extension of the infiltrated connective tissue (BC-aICT) measured 1.53 ± 0.91 mm for LI and 0.60 ± 0.34 mm for NLI, describing for the LI a larger area of bone that is in contact with infiltrated connective tissue. The mean distance between the margin of the peri-implant mucosa and the bottom of the bone defect (PM-BD) was 3.30 ± 1.37 mm for LI and 2.14 ± 0.37 mm for NLI, resulting in an increase of 65% in LI when compared to NLI. The mean height of the peri-implant mucosa in relation to the bone crest (PM-BC) was 1.96 ± 1.32 mm for LI and 1.80 ± 0.56 mm for NLI. The mean vertical distance between the margin of the peri-implant mucosa and the implant shoulder (PM-IS, e.g., the peri-implant mucosal recession) was 0.18 ± 1.38 mm for the LI and 1.34 ± 0.60 mm for the NLI. However, these values have to be interpreted taking into account both „true” mucosal recessions (negative values) and mucosal enlargements, resulting in false peri-implant pockets with positive values. In this regard, it is to be noted that NLI displayed only false pockets, thus positive values. The mean length of the barrier epithelium (PM-aJE) was on the average 1.66 ± 0.96 mm for LI and 1.05 ± 0.61 mm for NLI (approximately 63% higher in LI), in contradiction with the expected pathophysiology of the peri-implant lesion. The ICT mean apical extension was 3.49 ± 1.41 mm in LI compared to 2.30 ± 0.90 mm for NLI (PM-aICT), representing 66% more soft-tissue surrounding the implants in LI, due to heavily infiltrated connective tissue. The connective tissue located between the epithelium and the bone defect (e.g., the connective attachment, aJE-BD) had a mean height of 1.63 ± 2.03 mm for LI and 1.08 ± 0.53 mm for NLI, being obviously more abundant in LI. Finally, the distance between the most apical extension of the submarginal biofilm (interposed between the implant and the pocket epithelium) and the bottom of the bone defect (aPlaque-BD) measured 2.24 ± 0.61 mm for LI and 2.43 ± 0.23 mm for NLI. The mean value of the area occupied by the infiltrated connective tissue was 3.51 ± 2.49 mm^2^ for LI and 1.2 ± 0.62 mm^2^ for NLI (this is also reflected in IS-BD and BC-BD mean results). The mean value of the angle between the IS-BD and BD-BC segments was identical-51.71° ± 11.12 for LI and 51.75° ± 11.32 for NLI, despite the fact that in NLI the length of the buccal segment BC-BD was extremely reduced because of the severe resorption of the buccal cortical. 

### 3.3. CBCT Observations

Beam-hardening artifacts, dark streaks between implants and the photon starvation effect were only observed occasionally. These adverse effects and the buccal bone resorption were associated with a difficult depiction of the landmarks. 

In our experimental model, the mean values of buccal, oral, mesial and distal CBCT measurements were used to comparatively assess the bone loss between LI and NLI (Table 2).

When comparing LI and NLI on all four aspects, the supra-crestal component of the defect (SC) was on the average 2.32 ± 1.03 mm for LI and 1.09 ± 1.07 mm for NLI, suggesting that more bone was lost around ligated implants. The intrabony component of the defect (IC) measured 1.46 ± 1.00 mm for LI and 0.29 ± 0.29 mm for NLI, illustrating a crater-like defect around LI. The mean dimension of the defect width (DW) was 1.53 ± 0.84 mm for LI and 0.33 ± 0.32 mm for NLI describing a wider defect for LI. Finally, the mean of the angle between the IS-BD and BD-BC segments measured on all four aspects was 42.23° ± 21.95 for LI and 33.43° ± 28.62 for NLI.

A separate analysis of the mean values of mesial and distal CBCT measurements was effectuated for LI and NLI (Table 2). It is shown that the LI experienced more bone loss when compared to the supra-crestal component of the defect (SC) that was, on average, 2.31 ± 0.60 mm for LI and 0.92 ± 0.88 mm for NLI. The intrabony component of the defect (IC) was in mean 1.54 ± 0.97 mm for LI and 0.41 ± 0.32 mm for NLI, illustrating a pronounced vertical bone defect in LI. The mean dimension of the defect width (DW) was 1.58 ± 0.95 mm for LI and 0.28 ± 0.18 mm for NLI, describing a wider defect for LI that extends to the neighboring non-ligated implants. Finally, the mean of the angle between the IS-BD and BD-BC segments measured on the mesial and distal aspects was 48.41° ± 21.85 for LI and 26.4° ± 19.02 for NLI, describing a wider defect for LI.

Moreover, a separate analysis of the mean values of buccal and oral CBCT measurements was effectuated for LI and NLI (Table 2). Most of the mean values seemed to correlate with the corresponding histometric measurements. The mean value of the supra-crestal component of the defect (SC) measured on CBCT was 2.33 ± 1.41 mm for LI and 1.26 ± 1.35 mm for NLI, while the corresponding histometric mean value IS-BC measured 1.78 ± 1.31 mm for LI and 0.71 ± 0.63 mm for NLI. The mean value of the infra-crestal component of the defect (IC) measured on CBCT was on average 1.39 ± 1.12 mm for LI and 0.17 ± 0.25 mm, while the corresponding histometric mean value BC-BD was 1.33 ± 0.85 mm for LI and 0.33 ± 0.39 mm for NLI. The mean value of the defect width (DW) for LI measured on CBCT was on average 1.48 ± 0.81 mm for LI and 0.38 ± 0.44 mm for NLI, while the corresponding histometric mean value imp-BC was 1.70 ± 0.56 mm for NLI and 0.39 ± 0.38 mm for LI. Finally, the mean of the angle between the IS-BD and BD-BC segments for LI measured on CBCT 36.05° ± 22.16 and 40.47° ± 37.65 for NLI and the corresponding histometric mean value was 51.71° ± 11.12 and for LI and 51.75° ± 11.32 for NLI.

## 4. Discussion

Our experimental setting tried to reproduce the clinical reality of several adjacent similar implants but with different magnitudes of peri-implant involvement, while acknowledging the distance between the implants. The current study presents preliminary data aimed to confirm the influence of ligatured implants on adjacent non-ligatured implants, the latter presenting with naturally occurring mucositis and subsequent peri-implantitis as well—a situation encountered in numerous clinical situations. The study uses an animal dog model to study the experimentally induced peri-implantitis. For this purpose, the dog model was originally advocated by several authors [13,23,45], since these animals are preferentially used for studies conducted under compromised oral conditions [46] and because of the adequate jawbone anatomy for placement of dental implants [27]. In our model implants with various diameters were used to fit the actual width of the residual edentulous crest and to maintain a constant ratio of the distances to both corticals. This was also based on recent studies showing that bone loss increased with shorter and wider implants, however there were no significant differences in crestal bone loss for the tested implants regarding different diameters and lengths of implants [47,48,49,50]. 

In the above-mentioned study models, ligatures of cotton, silk or metal [51] were placed around properly integrated implants and forced into a submarginal position. Ligature placement compromised the mucosal attachment to the implant and promoted the buildup of plaque [14,23]. It was noted that inflammatory lesions rapidly formed, and bone loss occurred [13,23,52,53]. Unlike most of the aforementioned studies, where the ligatures were removed after the desired amount of tissue destruction was achieved, in our study ligatures were kept in contact with the peri-implant tissue during the whole experimental period, similar only to a more recent study design [42]. In our model, five months post-implantation (two months post implant exposure), to accelerate the progression of the initial lesions, cotton ligatures were placed in a submarginal position around the neck of implant No.1, two months later at implant No.3 and, after another two months, at implant No.5 (Figure 4). Two intermediate implants (No.2 and No.4) were left without ligatures, to compare the tissue breakdown between ligature-induced and spontaneous peri-implantitis in alternatively ligated implants. Additionally, the ligatures were placed at different timepoints, so that the progression in time of the lesions could be observed. After ligature-induced plaque accumulated on the implant abutment, the following loss of epithelial sealing [23] allowed the plaque front to continue to migrate apically undisturbed. However, generating „true” early peri-implant defects without the mechanical trauma of ligature placement was hypothesized in 2014 [25] and was demonstrated very recently [26]. To complement this approach, which separates the onset and progression of peri-implantitis on implants with different levels of plaque control, the present study addressed the influence of subsequently ligatured implants on adjacent non-ligatured implants in the same hemimandible in an orally compromised canine model that facilitated naturally occurring mucositis and subsequent peri-implantitis due to heavy, long-time, unrestricted dental plaque accumulation [26]. 

A recent study found that the number of adjacent implants is associated in humans with peri-implant disease, and this may be attributed to difficulties in performing adequate oral hygiene around multi-unit or splinted restorations, typically used in cases with multiple implants per patient [54]. This is partially reproduced by the serial placement of implants in our experimental study.

In our study, the histological examination of peri-implantitis sites disclosed that the lesion occurred not only in the mucosa but also frequently extended into the marrow space of the surrounding bone tissue, in line with previous studies [52]. The large inflammatory cell infiltrate and the presence of „open wound” characteristics of the pocket epithelium [10] (i.e., the epithelium covering less than the overall lesion dimension, insufficient to demarcate the entire inflammatory infiltrate from the biofilm challenge in the pocket area) was observed in our study as well. 

The ligature-induced marginal bone loss observed in this study is in agreement with data previously reported in a study that obtained similar results in the first two months after ligature placement [18]. 

Although implants 1, 3 and 5 were ligated at different points, similar results were noted. This means that, in our study, a similar pattern and speed of bone resorption occurred at the ligated implants. Although the mentioned mean results were similar, the difference was seen on the buccal aspect. This observation seems to be in accordance with Monje et al. [55], affirming that bone loss evolved more rapidly and more aggressively at the buccal sites versus the lingual counterparts. Additionally, it needs to be taken into consideration that the older lesions seemed to be self-limiting, despite the presence of the ligatures. This seems to be in accordance with the observations of Marinello et al. [13] that ligature-induced peri-implant lesions older than two months appear “inactive” and may become encapsulated, even after the removal of the ligatures. 

In both NLI implants, it was noted that the buccal plate underwent an almost complete resorption, up to the point that the segment BD-BC, which forms the angle between IS-BD and BC-BD, was reduced to less than 0.5 mm, making this particular angle measurable only under high magnifications. The above-mentioned buccal bone loss is in agreement with the CBCT observations of Monje et al., both on experimental peri-implantitis in dogs [55] and on human spontaneous peri-implantitis defects [31]. The almost complete loss of the buccal plate in NLI may be attributable to the original placement of the implants closer to the buccal aspect of the bone, in a prosthetic-driven position aimed to mimic the real clinical situation. In our study, the mean distance between the implant shoulder and bottom of the bone defect (IS-BD) revealed an advanced bone loss around LI due to the presence of ligatures. This artificially created bone defect does not mimic naturally occurring human peri-implantitis when compared to NLI.

In their experiment, Martins et al. [26] allowed for the establishment of a considerable amount of plaque prior to (16 weeks) and post-implant placement (17 weeks). In that study, the mean value of the distance measured from the mucosal margin to the most apical limit of the inflammatory infiltrate for implants without plaque control is in agreement with our corresponding histological measurement PM-aICT. According to this author, this could signify an ongoing soft tissue inflammation (peri-implant mucositis) that is mandatory preceding the peri-implantitis.

The mean distance between the margin of the peri-implant mucosa and bottom of the bone defect (PM-BD) resulted in an increase of 65% in LI when compared to NLI, suggesting that an intense inflammation occurred in a short period of time, so that tissues did not have time to remodulate. The mean height of the peri-implant mucosa in relation to the bone crest (PM-BC) was 1.96 ± 1.32 mm for LI and 1.80 ± 0.56 mm for NLI, possibly suggesting that the height of the peri-implant mucosa remains constant although the bone support is progressively being lost. 

Finally, the distance measured between the most apical extension of the submarginal biofilm (interposed between the implant and the pocket epithelium) and the bottom of the bone defect (aPlaque-BD), apparently was maintaining a constant distance between the apical extension of the plaque and the bottom of the bone defect, despite the presence of ligatures as a mean of accumulating plaque.

The histometric measurements with CBCT correspondents are SC, IC, DW, the angle made by IS-BD and BD-BC, all of them measured on buccal and oral. The Wilcoxon test showed that this difference was not statistically significant for global measurements. These results are in agreement with previous research that closely correlated CBCT analysis with the corresponding histological data [40]. The mesial and distal aspects of each implant where furthermore included in the CBCT analysis to compare the tissue losses between the subsequent moments of ligation, the mean values of the measurements on the mesial and distal aspects of the implants were used to evaluate the influence of LI on neighboring NLI. The distance between implants was indicated earlier to contribute to the overlapping and broadening of adjacent defects, thus influencing the final peri-implant defect configuration if the implants have been inserted too near to each other [25]. In the present study, the recommended minimal inter-implant distance of 3 mm (measured at the implant-abutment level) was respected [29]. This wide proximity between the implants in our research did not prevent the individual development of peri-implant defects and did not subvert their natural development, as shown by CBCT imaging. When comparing CBCT measurements on the mesial and distal aspect of the implants, the LI group had higher values than the NLI group. The present study found that peri-implantitis appeared to progress to a greater extent in LI implants than in NLI. These findings may be attributable to the vicinity of implant No. 1 and implant No. 5 to the full unaffected bone contours with no teeth or other implants in vicinity and thus the bone loss was less present.

The present pilot study had several limitations. One of them is the number of implants used, limited to a single experimental animal, in accordance with the most recent animal ethical recommendations. The sample size, although small, is normal for a pilot study, respecting the recent ARRIVE guidelines. Recent animal model studies reflect this tendency as well [52]. Another limitation addressed the fact that all implants were evaluated under unloaded conditions. It was previously reported that bone resorption was more pronounced when the implant was overloaded in the presence of plaque-induced peri-implantitis [56].

## 5. Conclusions

Within the limits of the present pilot study, the adequacy of the experimental dog model based on ligature-induced peri-implantitis was able to be successfully challenged by the non-ligature models of spontaneously occurring peri-implant inflammation, while meeting the requirements for experimental designs with a very small numbers of animals. Using the same study design, the evaluation of the influence of implants with severe peri-implantitis on adjacent implants revealed less than the expected tissue loss in the latter, with CBCT data complementing the histomorphometry. This finding might influence the choice of therapeutical approach when dealing with the various degrees of severity of the disease on adjacent implants. 

## Figures and Tables

**Table 1:** Histomorphometric results in LI and in NLI (means and SD).

Histometric Measurements		LI		NLI	
		Mean	SD	Mean	SD
Horizontal (mm)	imp-BC	1.70	0.56	0.39	0.38
Vertical Measurements (mm)	IS-BC	1.78	1.31	0.71	0.63
	IS-BD	3.12	0.58	0.79	0.55
	BC-BD	1.33	0.85	0.33	0.39
	BC-aICT	1.53	0.91	0.60	0.34
	PM-BC	1.96	1.32	1.80	0.56
	PM-IS	0.18	1.38	1.34	0.60
	PM-aJE	1.66	0.96	1.05	0.61
	PM-BD	3.30	1.37	2.14	0.37
	PM-aICT	3.49	1.41	2.30	0.90
	aJE-BD	1.63	2.03	1.08	0.53
	aPlaque-BD	2.24	0.61	2.43	0.23
Angle (degrees)	IS-BD BD-BC	51.71	11.12	51.75	11.32
Area (mm^2^)	ICT	3.51	2.49	1.2	0.62

**Table 2:** CBCT measurements of buccal, oral, mesial and distal aspects, of mesial and distal aspects and of buccal and oral aspects in LI and NLI (means and SD).

CBCT Measurements	Buccal, Oral, Mesial and Distal Aspects				Mesial and Distal Aspects				Buccal and Oral Aspects			
	LI		NLI		LI		NLI		LI		NLI	
	Mean	SD	Mean	SD	Mean	SD	Mean	SD	Mean	SD	Mean	SD
SC (mm)	2.32	1.03	1.09	1.07	2.31	0.60	0.92	0.88	2.33	1.41	1.26	1.35
IC (mm)	1.46	1.00	0.29	0.29	1.54	0.97	0.41	0.32	1.39	1.12	0.17	0.25
DW (mm)	1.53	0.84	0.33	0.32	1.58	0.95	0.28	0.18	1.48	0.81	0.38	0.44
Angle between IS-BD and BD-BC (degrees)	42.23	21.95	33.43	28.62	48.41	21.85	26.4	19.02	36.05	22.16	40.47	37.65

## Data Availability

Data are available from the corresponding author upon reasonable request.

## References

[1] Albouy J.P., Abrahamsson I., Persson L.G., Berglundh T. (2008). Spontaneous progression of peri-implantitis at different types of implants. An experimental study in dogs. I: Clinical and radiographic observations. Clin. Oral Implant. Res..

[2] Berglundh T., Persson L., Klinge B. (2002). A systematic review of the incidence of biological and technical complications in implant dentistry reported in prospective longitudinal studies of at least 5 years. J. Clin. Periodontol..

[3] Fransson C., Lekholm U., Jemt T., Berglundh T. (2005). Prevalence of subjects with progressive bone loss at implants. Clin. Oral. Implant. Res..

[4] Fransson C., Wennström J., Berglundh T. (2008). Clinical characteristics at implants with a history of progressive bone loss. Clin. Oral Implant. Res..

[5] Roos-Jansåker A.M., Lindahl C., Renvert H., Renvert S. (2006). Nine- to fourteen-year follow-up of implant treatment. Part II: Presence of peri-implant lesions. J. Clin. Periodontol..

[6] Berglundh T., Zitzmann N.U., Donati M. (2011). Are peri-implantitis lesions different from periodontitis lesions?. J. Clin. Periodontol..

[7] Heitz-Mayfield L.J. (2008). Peri-implant diseases: Diagnosis and risk indicators. J. Clin. Periodontol..

[8] Schwarz F., Derks J., Monje A., Wang H.L. (2018). Peri-implantitis. J. Periodontol..

[9] Weiss R., Read-Fuller A. (2019). Cone Beam Computed Tomography in Oral and Maxillofacial Surgery: An Evidence-Based Review. Dent. J..

[10] Albouy J.P., Abrahamsson I., Persson L.G., Berglundh T. (2009). Spontaneous progression of ligatured induced peri-implantitis at implants with different surface characteristics. An experimental study in dogs II: Histological observations. Clin. Oral Implant. Res..

[11] Carcuac O., Abrahamsson I., Charalampakis G., Berglundh T. (2015). The effect of the local use of chlorhexidine in surgical treatment of experimental peri-implantitis in dogs. J. Clin. Periodontol..

[12] Ericsson I., Lindhe J., Rylander H., Okamoto H. (1975). Experimental periodontal breakdown in the dog. Scand. J. Dent. Res..

[13] Marinello C.P., Berglundh T., Ericsson I., Klinge B., Glantz P.O., Lindhe J. (1995). Resolution of ligature-induced peri-implantitis lesions in the dog. J. Clin. Periodontol..

[14] Wetzel A.C., Vlassis J., Caffesse R.G., Hämmerle C.H., Lang N.P. (1999). Attempts to obtain re-osseointegration following experimental peri-implantitis in dogs. Clin. Oral Implant. Res..

[15] Directive 2010/63/EU of the European Parliament and of the Council of 22 September 2010 on the Protection of Animals Used for Scientific Purposes. https://eur-lex.europa.eu/eli/dir/2010/63.

[16] Percie du Sert N., Hurst V., Ahluwalia A., Alam S., Avey M.T., Baker M., Browne W.J., Clark A., Cuthill I.C., Dirnagl U. (2020). The ARRIVE guidelines 2.0: Updated guidelines for reporting animal research. Br. J. Pharmacol..

[17] Kilkenny C., Browne W., Cuthill I.C., Emerson M., Altman D.G. (2010). NC3Rs Reporting Guidelines Working Group. Animal research: Reporting in vivo experiments: The ARRIVE guidelines. Br. J. Pharmacol..

[18] Zitzmann N.U., Berglundh T., Ericsson I., Lindhe J. (2004). Spontaneous progression of experimentally induced periimplantitis. J. Clin. Periodontol..

[19] Berglundh T., Gotfredsen K., Zitzmann N.U., Lang N.P., Lindhe J. (2007). Spontaneous progression of ligature induced peri-implantitis at implants with different surface roughness: An experimental study in dogs. Clin. Oral Implant. Res..

[20] Albouy J.P., Abrahamsson I., Persson L.G., Berglundh T. (2011). Implant surface characteristics influence the outcome of treatment of peri-implantitis: An experimental study in dogs. J. Clin. Periodontol..

[21] Albouy J.P., Abrahamsson I., Berglundh T. (2012). Spontaneous progression of experimental peri-implantitis at implants with different surface characteristics: An experimental study in dogs. J. Clin. Periodontol..

[22] Reinedahl D., Chrcanovic B., Albrektsson T., Tengvall P., Wennerberg A. (2018). Ligature-Induced Experimental Peri-Implantitis-A Systematic Review. J. Clin. Med..

[23] Lindhe J., Berglundh T., Ericsson I., Liljenberg B., Marinello C. (1992). Experimental breakdown of peri-implant and periodontal tissues. A study in the beagle dog. Clin. Oral Implant. Res..

[24] Albrektsson T., Canullo L., Cochran D., De Bruyn H. (2016). “Peri-Implantitis”: A Complication of a Foreign Body or a Man-Made “Disease”. Facts and Fiction. Clin. Implant Dent. Relat. Res..

[25] Martins O., Ramos J.C., Baptista I.P., Dard M.M. (2014). The dog as a model for peri-implantitis: A review. J. Investig. Surg..

[26] Martins O., Ramos J.C., Mota M., Dard M., Viegas C., Caramelo F., Nogueira C., Gonçalves T., Baptista I.P. (2020). Evaluation of a novel dog animal model for peri-implant disease: Clinical, radiographic, microbiological and histological assessment. Clin. Oral Investig..

[27] Battula S., Lee J.W., Wen H.B., Papanicolaou S., Collins M., Romanos G.E. (2015). Evaluation of Different Implant Designs in a Ligature-Induced Peri-implantitis Model: A Canine Study. Int. J. Oral Maxillofac. Implant..

[28] Baron M., Haas R., Dörtbudak O., Watzek G. (2000). Experimentally induced peri-implantitis: A review of different treatment methods described in the literature. Int. J. Oral Maxillofac. Implant..

[29] Tarnow D.P., Cho S.C., Wallace S.S. (2000). The effect of inter-implant distance on the height of inter-implant bone crest. J. Periodontol..

[30] Albrektsson T., Buser D., Sennerby L. (2012). Crestal Bone Loss and Oral implants. Clin. Implant Dent. Relat. Res..

[31] Monje A., Insua A., Wang H.L. (2019). Understanding Peri-Implantitis as a Plaque-Associated and Site-Specific Entity: On the Local Predisposing Factors. J. Clin. Med..

[32] Jalaluddin M., El-Patal M.A.-E., Barakat I., Alzahrani K.M., Fathi A. (2021). Elgendy Abdelraheem Ramadan. Evaluation of Periodontal Disease in Teeth Adjacent to Implant with Peri-Implantitis–A Clinical Study. Eur. J. Mol. Clin. Med..

[33] Hashim D., Cionca N. (2020). A Comprehensive Review of Peri-implantitis Risk Factors. Curr. Oral Health Rep..

[34] Kumar P.S., Dabdoub S.M., Hegde R., Ranganathan N., Mariotti A. (2018). Site-level risk predictors of peri-implantitis: A retrospective analysis. J. Clin. Periodontol..

[35] Wada M., Mameno T., Otsuki M., Kani M., Tsujioka Y., Ikebe K. (2021). Prevalence and risk indicators for peri-implant diseases: A literature review. Jpn. Dent. Sci. Rev..

[36] Passoni B.B., Dalago H.R., Schuldt Filho G., Oliveira de Souza J.G., Benfatti C.A., Magini Rde S., Bianchini M.A. (2014). Does the number of implants have any relation with peri-implant disease?. J. Appl. Oral Sci..

[37] Papalou I., Vagia P., Cakir A., Tenenbaum H., Huck O., Davideau J.L. (2022). Influence of Periodontitis, Implant, and Prosthesis Characteristics on the Peri-Implant Status: A Cross-Sectional Study. Int. J. Dent..

[38] Schwarz F., Sahm N., Mihatovic I., Golubovic V., Becker J. (2011). Surgical therapy of advanced ligature-induced peri-implantitis defects: Cone-beam computed tomographic and histological analysis. J. Clin. Periodontol..

[39] von Wilmowsky C., Moest T., Nkenke E., Stelzle F., Schlegel K.A. (2014). Implants in bone: Part II. Research on implant osseointegration: Material testing, mechanical testing, imaging and histoanalytical methods. Oral Maxillofac. Surg..

[40] Golubovic V., Mihatovic I., Becker J., Schwarz F. (2012). Accuracy of conebeam computed tomography to assess the configuration and extent of ligature-induced peri-implantitis defects. A pilot study. Oral Maxillofac. Surg..

[41] Zechner W., Kneissel M., Kim S., Ulm C., Watzek G., Plenk H. (2004). Histomorphometrical and clinical comparison of submerged and nonsubmerged implants subjected to experimental peri-implantitis in dogs. Clin. Oral Implant. Res..

[42] Huang B., Zhang L., Xu L., Zhu W., Witek L., Tovar N., Coelho P.G., Meng H. (2018). Effect of implant placement depth on the peri-implant bone defect configurations in ligature-induced peri-implantitis: An experimental study in dogs. Med. Oral Patol. Oral Cir. Bucal..

[43] Donath K., Breuner G. (1982). A method for the study of undecalcified bones and teeth with attached soft tissues. The Säge-Schliff (sawing and grinding) technique. J. Oral Pathol..

[44] de Chaumont F., Dallongeville S., Chenouard N., Hervé N., Pop S., Provoost T., Meas-Yedid V., Pankajakshan P., Lecomte T., Le Montagner Y. (2012). Icy: An open bioimage informatics platform for extended reproducible research. Nat. Methods.

[45] Schou S., Holmstrup P., Stoltze K., Hjørting-Hansen E., Kornman K.S. (1993). Ligature-induced marginal inflammation around osseointegrated implants and ankylosed teeth. Clin. Oral Implant. Res..

[46] Blanc-Sylvestre N., Bouchard P., Chaussain C., Bardet C. (2021). Pre-Clinical Models in Implant Dentistry: Past, Present, Future. Biomedicines.

[47] Kowalski J., Lapinska B., Nissan J., Lukomska-Szymanska M. (2021). Factors Influencing Marginal Bone Loss around Dental Implants: A Narrative Review. Coatings.

[48] Winkler S., Morris H.F., Ochi S. (2000). Implant survival to 36 months as related to length and diameter. Ann. Periodontol..

[49] Chung D.M., Oh T.J., Lee J., Misch C.E., Wang H.L. (2007). Factors affecting late implant bone loss: A retrospective analysis. J. Prosthet. Dent..

[50] Monje A., Suarez F., Galindo-Moreno P., García-Nogales A., Fu J.H., Wang H.L. (2014). A systematic review on marginal bone loss around short dental implants (<10 mm) for implant-supported fixed prostheses. Clin. Oral Implant. Res..

[51] Lin X., Liu T., Wu G., Zheng Y., Wismeijer D., Liu Y. (2017). Peri-implantitis Induced by Stainless Steel Ligature in Beagle Dogs. Int. J. Periodontics Restor. Dent..

[52] Persson L.G., Ericsson I., Berglundh T., Lindhe J. (2001). Osse integration following treatment of peri-implantitis and replacement of implant components. An experimental study in the dog. J. Clin. Periodontol..

[53] Persson L.G., Araújo M.G., Berglundh T., Gröndahl K., Lindhe J. (1999). Resolution of peri-implantitis following treatment. An experimental study in the dog. Clin. Oral Implant Res..

[54] Rinke S., Nordlohne M., Leha A., Renvert S., Schmalz G., Ziebolz D. (2020). Risk indicators for mucositis and peri-implantitis: Results from a practice-based cross-sectional study. J. Periodontal Implant Sci..

[55] Monje A., Insua A., Rakic M., Nart J., Moyano-Cuevas J.L., Wang H.L. (2018). Estimation of the diagnostic accuracy of clinical parameters for monitoring peri-implantitis progression: An experimental canine study. J. Periodontol..

[56] Chambrone L., Chambrone L.A., Lima L.A. (2010). Effects of occlusal overload on peri-implant tissue health: A systematic review of animal-model studies. J. Periodontol..

